# Differentially mutated subnetworks discovery

**DOI:** 10.1186/s13015-019-0146-7

**Published:** 2019-03-30

**Authors:** Morteza Chalabi Hajkarim, Eli Upfal, Fabio Vandin

**Affiliations:** 1 Biotech Research and Innovation Centre, University of Copenhagen, Copenhagen, Denmark; 2 Department of Computer Science, Brown University, Providence, RI USA; 3 Department of Information Engineering, University of Padova, Padova, Italy

**Keywords:** Network analysis, Somatic mutations, Differential analysis

## Abstract

**Problem:**

We study the problem of identifying differentially mutated subnetworks of a large gene–gene interaction network, that is, subnetworks that display a significant difference in mutation frequency in two sets of cancer samples. We formally define the associated computational problem and show that the problem is NP-hard.

**Algorithm:**

We propose a novel and efficient algorithm, called DAMOKLE, to identify differentially mutated subnetworks given genome-wide mutation data for two sets of cancer samples. We prove that DAMOKLE identifies subnetworks with statistically significant difference in mutation frequency when the data comes from a reasonable generative model, provided enough samples are available.

**Experimental results:**

We test DAMOKLE on simulated and real data, showing that DAMOKLE does indeed find subnetworks with significant differences in mutation frequency and that it provides novel insights into the molecular mechanisms of the disease not revealed by standard methods.

## Introduction

The analysis of molecular measurements from large collections of cancer samples has revolutionized our understanding of the processes leading to a tumour through somatic mutations, changes of the DNA appearing during the lifetime of an individual [1]. One of the most important aspects of cancer revealed by recent large cancer studies is *inter-tumour genetic heterogeneity*: each tumour presents hundreds-thousands mutations and no two tumours harbour the same set of DNA mutations [2].

One of the fundamental problems in the analysis of somatic mutations is the identification of the handful of *driver mutations* (i.e., mutations related to the disease) of each tumour, detecting them among the thousands or tens of thousands that are present in each tumour genome [3]. Inter-tumour heterogeneity renders the identification of driver mutations, or of driver genes (genes containing driver mutations), extremely difficult, since only few genes are mutated in a relatively large fraction of samples while most genes are mutated in a low fraction of samples in a cancer cohort [4].

Recently, several analyses (e.g, [5, 6]) have shown that interaction networks provide useful information to discover driver genes by identifying groups of interacting genes, called *pathways*, in which each gene is mutated at relatively low frequency while the entire group has one or more mutations in a significantly large fraction of all samples. Several network-based methods have been developed to identify groups of interacting genes mutated in a significant fraction of tumours of a given type and have been shown to improve the detection of driver genes compared to methods that analyze genes in isolation [5, 7–9].

The availability of molecular measurements in a large number of samples for different cancer types have also allowed *comparative* analyses of mutations in cancer [5, 10, 11]. Such analyses usually analyze large cohorts of different cancer types as a whole employing methods to find genes or subnetworks mutated in a significant fraction of tumours in *one* cohort, and also analyze each cancer type individually, with the goal to identify:

1. pathways that are common to various cancer types;
2. pathways that are specific to a given cancer type.

For example, [5] analyzed 12 cancer types and identified subnetworks (e.g., a TP53 subnetwork) mutated in most cancer types as well as subnetworks (e.g., a MHC subnetwork) enriched for mutations in one cancer type. In addition, comparative analyses may also be used for the identification of mutations of clinical relevance [12]. For example: comparing mutations in a patients that responded to a given therapy with mutations in patients (of the same cancer type) that did not respond to the same therapy may identify genes and subnetworks associated with response to therapy; comparing mutations in patients whose tumours metastasized with mutations in patients whose tumours did not metastasize may identify mutations associated with the insurgence of metastases.

**Fig. 1:**
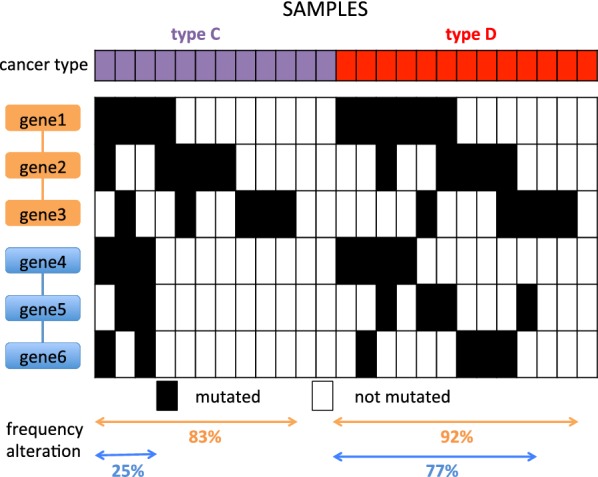
Identification of subnetworks with significant difference in mutation frequency in two set of samples ${\mathcal {C}}, {\mathcal {D}}$. The blue subnetwork is significantly more mutated in ${\mathcal {D}}$ than in ${\mathcal {C}}$, but it is not detected by methods that look for the most significantly mutated subnetworks in ${\mathcal {C}}$ or in ${\mathcal {D}}$ or in ${\mathcal {C}}\cup {\mathcal {D}}$, since the orange subnetwork is in each case mutated at much higher frequency

Pathways that are significantly mutated only in a specific cancer type may not be identified by analyzing one cancer type at the time or all samples together (Fig. 1), but, interestingly, to the best of our knowledge no method has been designed to *directly* identify sets of interacting genes that are significantly more mutated in a set of samples compared to another. The task of finding such sets is more complex than the identification of subnetworks significantly mutated in a set of samples, since subnetworks that have a significant difference in mutations in two sets may display relatively modest frequency of mutation in both set of samples, whose difference can be assessed as significant only by the joint analysis of both sets of samples.

### Related work

Several methods have been designed to analyze different aspects of somatic mutations in a large cohort of cancer samples in the context of networks. Some methods analyze mutations in the context of known pathways to identify the ones significantly enriched in mutations (e.g., [13]). Other methods combine mutations and large interaction networks to identify cancer subnetworks [5, 14, 15]. Networks and somatic mutations have also been used to prioritarize mutated genes in cancer [7, 8, 16–18] and for patients stratification [6, 19]. Some of these methods have been used for the identification of common mutation patterns or subnetworks in several cancer types [5, 10], but to the best of our knowledge no method has been designed to identify mutated subnetworks with a significant difference in two cohorts of cancer samples.

Few methods studied the problem of identifying subnetworks with significant differences in two sets of cancer samples using data other than mutations. [20] studied the problem of identifying optimally discriminative subnetworks of a large interaction network using gene expression data. Mall et al. [21] developed a procedure to identify statistically significant changes in the topology of biological networks. Such methods cannot be readily applied to find subnetworks with significant difference in mutation frequency in two sets of samples. Other related work use gene expression to characterize different cancer types: [22] defined a pathway-based score that clusters samples by cancer type, while [23] defined pathway-based features used for classification in various settings, and several methods [24–28] have been designed for finding subnetworks with differential gene expression.

### Our contribution

In this work we study the problem of finding subnetworks with frequency of mutation that is significantly different in two sets of samples. In particular, our contributions are fourfold. First, we propose a combinatorial formulation for the problem of finding subnetworks significantly more mutated in one set of samples than in another and prove that such problem is NP-hard. Second, we propose DifferentiAlly Mutated subnetwOrKs anaLysis in cancEr (DAMOKLE), a simple and efficient algorithm for the identification of subnetworks with a significant difference of mutation in two sets of samples, and analyze DAMOKLE proving that it identifies subnetworks significantly more mutated in one of two sets of samples under reasonable assumptions for the data. Third, we test DAMOKLE on simulated data, verifying experimental that DAMOKLE correctly identifies subnetworks significantly more mutated in a set of samples when enough samples are provided in input. Fourth, we test DAMOKLE on large cancer datasets comprising two cancer types, and show that DAMOKLE identifies subnetworks significantly associated with one of the two types which cannot be identified by state-of-the-art methods designed for the analysis of one set of samples.

## Methods and algorithms

This section presents the problem we study, the algorithm we propose for its solution, and the analysis of our algorithm. In particular, "Computational problem" section formalizes the computational problem we consider; "Algorithm" section presents DifferentiAlly Mutated subnetwOrKs anaLysis in cancEr (DAMOKLE), our algorithm for the solution of the computational problem; "Analysis of DAMOKLE" section describes the analysis of our algorithm under a reasonable generative model for mutations; "Statistical significance of the results" section presents a formal analysis of the statistical significance of subnetworks obtained by DAMOKLE; and "Permutation testing" section describes two permutation tests to assess the significance of the results of DAMOKLE for limited sample sizes.

### Computational problem

We are given measurements on mutations in *m* genes $\mathcal {G}=\{1,\dots ,m\}$ on two sets ${\mathcal {C}}=\{c_1,\dots ,c_{n_C}\},{\mathcal {D}}=\{d_1,\dots ,d_{n_D}\}$ of samples. Such measurements are represented by two matrices *C* and *D*, of dimension $m \times n_C$ and $m \times n_D$, respectively, where $n_C$ (resp., $n_D$) is the number of samples in ${\mathcal {C}}$ (resp., ${\mathcal {D}}$). $C(i,j)=1$ (resp., $D(i,j)=1$) if gene *i* is mutated in the *j*-th sample of ${\mathcal {C}}$ (resp., ${\mathcal {D}}$) and $C(i,j)=0$ (resp., $D(i,j)=0$) otherwise. We are also given an (undirected) graph $G=(V,E)$, where vertices $V = \{1,\dots ,m \}$ are genes and $(i,j) \in E$ if gene *i* interacts with gene *j* (e.g., the corresponding proteins interact).

Given a set of genes $S \subset \mathcal {G}$, we define the indicator function $c_{S}(c_i)$ with $c_{S}(c_i)=1$ if at least one of the genes of *S* is mutated in sample $c_i$, and $c_{S}(c_i)=0$ otherwise. We define $c_{S}(d_i)$ analogously. We define the *coverage *$c_{S}({\mathcal {C}})$* of S in *${\mathcal {C}}$ as the fraction of samples in ${\mathcal {C}}$ for which at least one of the genes in *S* is mutated in the sample, that is

$$\begin{aligned} c_{S}({\mathcal {C}}) = \frac{\sum _{i=1}^{n_C} c_{S}(c_i)}{n_C} \end{aligned}$$

and, analogously, define the *coverage *$c_{S}({\mathcal {D}})$* of* *S in *${\mathcal {D}}$ as $c_{S}({\mathcal {D}}) = \frac{\sum _{i=1}^{n_D} c_{S}(d_i)}{n_D}.$

We are interested in identifying sets of genes *S*, with $|S|\le k$, corresponding to connected subgraphs in *G* and displaying a *significant* difference in coverage between ${\mathcal {C}}$ and ${\mathcal {D}}$, i.e., with a high value of $|c_{S}({\mathcal {C}})-c_{S}({\mathcal {D}})|$. We define the *differential coverage* $dc_{S}({\mathcal {C}},{\mathcal {D}})$ as $dc_{S}({\mathcal {C}},{\mathcal {D}}) = c_{S}({\mathcal {C}})-c_{S}({\mathcal {D}}).$

In particular, we study the following computational problem.

The differentially mutated subnetworks discovery problem: given a value $\theta$ with $\theta \in [0,1]$, find all connected subgraphs *S* of *G* of size $\le k$ such that $dc_{S}({\mathcal {C}},{\mathcal {D}}) \ge \theta$.

Note that by finding sets that maximize $dc_{S}({\mathcal {C}},{\mathcal {D}})$ we identify sets with significantly more mutations in ${\mathcal {C}}$ than in ${\mathcal {D}}$, while to identify sets with significantly more mutations in ${\mathcal {D}}$ than in ${\mathcal {C}}$ we need to find sets maximizing $dc_{S}({\mathcal {D}},{\mathcal {C}})$. In addition, note that a subgraph *S* in the solution may contain genes that are not mutated in ${\mathcal {C}}\cup {\mathcal {D}}$ but that are needed for the connectivity of *S*.

We have the following.

#### **Theorem 1**

*The differentially mutated subnetworks discovery problem is NP-hard*.

#### *Proof*

The proof is by reduction from the connected maximum coverage problem [14]. In the connected maximum coverage problem we are given a graph *G* defined on a set $V=\{v_1,\dots ,v_n\}$ of *n* vertices, a family $\mathcal {P} = \{P_1,\dots ,P_n\}$ of subsets of a universe *I* (i.e., $P_i \in 2^{I}$), with $P_i$ being the subset of *I* *covered* by $v_i \in V$ and value *k*, and we want to find the subgraph $C^* = \{v_{i_1},\dots , v_{i_k}\}$ with *k* nodes of *G* that maximizes $|\cup _{j=1}^k P_{i_j}|$.

Given an instance of the connected maximum coverage problem, we define an instance of the differentially mutated subnetworks discovery problem as follows: the set $\mathcal {G}$ of genes corresponds to the set *V* of vertices of *G* in the connected maximum coverage problem, and the graph *G* is the same as in the instance of the maximum coverage instance; the set ${\mathcal {C}}$ is given by the set *I* and the matrix *C* is defined as $C_{i,j}=1$ if $i \in P_j$, while ${\mathcal {D}}=\emptyset$.

Note that for any subgraph *S* of *G*, the differential coverage $dc_D({\mathcal {C}},{\mathcal {D}})= c_{S}({\mathcal {C}}) - c_{S}({\mathcal {D}}) = c_{S}({\mathcal {C}})$ and $c_{S}({\mathcal {C}}) = |\cup _{g \in S} P_{g}|/|I|$. Since |*I*| is the same for all solutions, the optimal solution of the differentially mutated subnetworks discovery instance corresponds to the optimal solution to the connected maximum coverage instance, and viceversa. $\square$

### Algorithm

We now describe DifferentiAlly Mutated subnetwOrKs anaLysis in cancEr (DAMOKLE), an algorithm to solve the differentially mutated subnetworks discovery problem. DAMOKLE takes in input mutation matrices *C* and *D* for two sets ${\mathcal {C}}$, ${\mathcal {D}}$ of samples, a (gene–gene) interaction graph *G*, an integer $k>0$, and a real value $\theta \in [0,1]$, and returns subnetworks *S* of *G* with $\le k$ vertices and differential coverage $dc_{S}({\mathcal {C}},{\mathcal {D}}) \ge \theta$. Subnetworks reported by DAMOKLE are also *maximal* (no vertex can be added to *S* while maintaining the connectivity of the subnetwork, $|S| \le k$ *and* $dc_{S}({\mathcal {C}},{\mathcal {D}}) \ge \theta$). DAMOKLE is described in Algorithm 1. DAMOKLE starts by considering each edge $e=\{u,v\} \in E$ of *G* with differential coverage $dc_{\{u,v\}}({\mathcal {C}},{\mathcal {D}})\ge \theta /(k-1)$, and for each such *e* identifies subnetworks including *e* to be reported in output using Algorithm 2. 
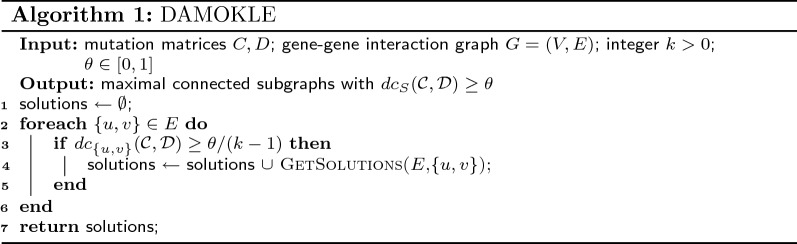


GetSolutions, described in Algorithm 2, is a recursive algorithm that, give a current subgraph *S*, identifies all maximal connected subgraphs $S', |S'| \le k$, containing *S* and with $dc_{S'}({\mathcal {C}},{\mathcal {D}}) \ge \theta$. This is obtained by expanding *S* one edge at the time and stopping when the number of vertices in the current solution is *k* or when the addition of no vertex leads to an increase in differential coverage $dc_{S}({\mathcal {C}},{\mathcal {D}})$ for the current solution *S*. In Algorithm 2, *N*(*S*) refers to the set of edges with exactly one vertex in the set *S*. 
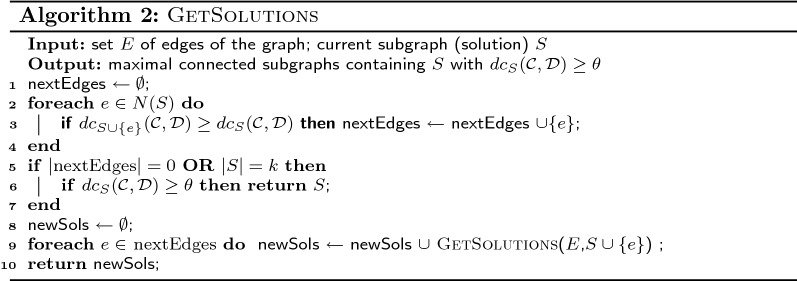


The motivation for design choices of DAMOKLE are provided by the results in the next section.

### Analysis of DAMOKLE

The design and analysis of DAMOKLE are based on the following generative model for the underlying biological process.

#### Model

For each gene $i \in \mathcal {G}=\{1,2,...,m\}$ there is an a-priori probability $p_i$ of observing a mutation in gene *i*. Let $H\subset \mathcal {G}$ be the connected subnetwork of up to *k* genes that is differentially mutated in samples of ${\mathcal {C}}$ w.r.t. samples of ${\mathcal {D}}$. Mutations in our samples are taken from two related distributions. In the “control” distribution *F* a mutation in gene *i* is observed with probability $p_i$ independent of other genes’ mutations. The second distribution $F_H$ is analogous to the distribution *F* but we condition on the event $E(H)=$“at least one gene in *H* is mutated in the sample”.

For genes not in *H*, all mutations come from distribution *F*. For genes in *H*, in a perfect experiment with no noise we would assume that samples in ${\mathcal {C}}$ are taken from $F_H$ and samples from ${\mathcal {D}}$ are taken from *F*. However, to model realistic, noisy data we assume that with some probability *q* the “true” signal for a sample is lost, that is the sample from ${\mathcal {C}}$ is taken from *F*. In particular, samples in ${\mathcal {C}}$ are taken with probability $1-q$ from $F_H$ and with probability *q* from *F*.

Let *p* be the probability that *H* has at least one mutation in samples from the control model *F*, $p= 1-\prod _{j\in H} (1-p_j)\approx \sum _{j\in H} p_j.$ Clearly, we are only interested in sets $H\subset \mathcal {G}$ with $p\ll 1$.

If we focus on individual genes, the probability gene *i* is mutated in a sample from ${\mathcal {D}}$ is $p_i$, while the probability that it is mutated in a sample from ${\mathcal {C}}$ is $\frac{(1-q)p_i}{1-\prod _{j\in H} (1-p_j)}+qp_i.$ Such a gap may be hard to detect with a small number of samples. On the other hand, the probability of *E*(*H*) (i.e., of at least one mutation in the set *H*) in a sample from ${\mathcal {C}}$ is $(1-q) +q(1-\prod _{j\in H} (1-p_j)) = 1-q + qp$, while the probability of *E*(*H*) in a sample from ${\mathcal {D}}$ is $1-\prod _{j\in H} (1-p_j) = p$ which is a more significant gap, when $p \ll 1.$

The efficiency of DAMOKLE is based on two fundamental results. First we show that it is sufficient to start the search only in edges with relatively high differential coverage.

##### **Proposition 1**

*If* $dc_{S}({\mathcal {C}},{\mathcal {D}}) \ge \theta,$ *then, in the above generating model, with high probability (asymptotic in* $n_C$ *and* $n_D$ *)there exist an edge* $e \in S$ *such that* $dc_{\{e\}}({\mathcal {C}},{\mathcal {D}}) \ge (\theta -\epsilon )/(k-1),$ *for any* $\epsilon >0.$

##### *Proof*

For a set of genes $S'\subset \mathcal {G}$ and a sample $z\in {\mathcal {C}} \cup {\mathcal {D}}$, let $Count(S',z)$ be the number of genes in $S'$ mutated in sample *z*. Clearly, if for all $z\in {\mathcal {C}} \cup {\mathcal {D}}$, we have $Count(S,z)=1$, i.e. each sample has no more than one mutation in *S*, then

$$\begin{aligned} dc_{S}({\mathcal {C}},{\mathcal {D}})=\, & {} c_{S}({\mathcal {C}})-c_{S}({\mathcal {D}}) =\,\frac{\sum _{i=1}^{n_C} c_{S}(c_i)}{n_C} - \frac{\sum _{i=1}^{n_D} c_{S}(d_i)}{n_D} \\=\, & {} \frac{\sum _{i=1}^{n_C} \sum _{j\in S} Count (\{j\}, c_i)}{n_C} - \frac{\sum _{i=1}^{n_D} \sum _{j\in S} Count(\{j\},d_i)}{n_D} \\= \,& {} \sum _{j\in S} \left( \frac{\sum _{i=1}^{n_C} Count (\{j\}, c_i)}{n_C} - \frac{\sum _{i=1}^{n_D} Count(\{j\},d_i)}{n_D} \right) \\\ge & {} \theta . \end{aligned}$$

Thus, there is a vertex $j^*=\arg \max _{j\in S} \left( \frac{\sum _{i=1}^{n_C} Count (\{j\}, c_i)}{n_C} - \frac{\sum _{i=1}^{n_D} Count(\{j\},d_i)}{n_D} \right)$ such that $dc_{\{j^*\}}({\mathcal {C}},{\mathcal {D}}) =\frac{\sum _{i=1}^{n_C} Count (\{j^*\}, c_i)}{n_C} - \frac{\sum _{i=1}^{n_D} Count(\{j^*\},d_i)}{n_D} \ge \theta /k.$

Since the set of genes *S* is connected, there is an edge $e=(j^*, \ell )$ for some $\ell \in S$. For that edge,

$$\begin{aligned} dc_{\{e \}}({\mathcal {C}},{\mathcal {D}}) \ge \frac{\theta -dc_{\{\ell \}}({\mathcal {C}},{\mathcal {D}})}{k-1} +dc_{\{\ell \}}({\mathcal {C}},{\mathcal {D}}) \ge \frac{\theta }{k-1}. \end{aligned}$$

For the case when the assumption $Count(S,z)=1$ for all $z \in {\mathcal {C}}\cup {\mathcal {D}}$ does not hold, let

$$\begin{aligned} Mul(S, {\mathcal {C}},{\mathcal {D}})= & {} \frac{\sum _{i=1}^{n_C} \sum _{j\in S} Count (\{j\}, c_i)}{n_C} - \frac{\sum _{i=1}^{n_C} c_{S}(c_i)}{n_C} \\&+ \frac{\sum _{i=1}^{n_D} Count(\{j\},d_i)}{n_D} -\frac{\sum _{i=1}^{n_D} c_{S}(d_i)}{n_D}. \end{aligned}$$

Then

$$\begin{aligned} \sum _{j\in S} \left( \frac{\sum _{i=1}^{n_C} Count (\{j\}, c_i)}{n_C} - \frac{\sum _{i=1}^{n_D} Count(\{j\},d_i)}{n_D} \right) - Mul(S, {\mathcal {C}},{\mathcal {D}}) \ge \theta \end{aligned}$$

and

$$\begin{aligned} dc_{\{e \}}({\mathcal {C}},{\mathcal {D}})\ge \frac{\theta +Mul(S, {\mathcal {C}},{\mathcal {D}}) }{k-1}. \end{aligned}$$

Since the probability of having more than one mutation in *S* in a sample from ${\mathcal {C}}$ is at least as high as from a sample from ${\mathcal {D}}$, we can normalize (similar to the proof of Theorem 2 below) and apply Hoeffding bound (Theorem 4.14 in [29]) to prove that

$$\begin{aligned} Prob(Mul(S, {\mathcal {C}},{\mathcal {D}}) < -\epsilon )\le 2e^{-2\epsilon ^2 n_C n_D/(n_C+n_D)}. \end{aligned}$$

$$\square$$

The second result motivates the choice, in Algorithm 2, of adding only edges that increase the score of the current solution (and to stop if there is no such edge).

##### **Proposition 2**

*If subgraph* *S* *can be partitioned as* $S= S' \cup \{j\} \cup S'',$ *and* $dc_{\mathcal {S'}\cup \{j\}}({\mathcal {C}},{\mathcal {D}})< dc_{\mathcal {S'}}({\mathcal {C}},{\mathcal {D}})- p p_j,$ *then with high probability (asymptotic in* $n_{{\mathcal {D}}}$ *)* $dc_{S \setminus \{j\}}({\mathcal {C}},{\mathcal {D}}) > dc_{S}({\mathcal {C}},{\mathcal {D}}).$

##### *Proof*

We first observe that if each sample in ${\mathcal {D}}$ has no more than 1 mutation in *S* then $dc_{\mathcal {S'}\cup \{j\}}({\mathcal {C}},{\mathcal {D}})< dc_{\mathcal {S'}}({\mathcal {C}},{\mathcal {D}})$ implies that $dc_{\{j\}}({\mathcal {C}},{\mathcal {D}})<0$, and therefore, under this assumption, $dc_{S \setminus \{j\}}({\mathcal {C}},{\mathcal {D}}) > dc_{S}({\mathcal {C}},{\mathcal {D}})$.

To remove the assumption that a sample has no more than one mutation in *S*, we need to correct for the fraction of samples in ${\mathcal {D}}$ with mutations both in *j* and $S''$. With high probability (asymptotic in $n_D$) this fraction is bounded by $pp_j +\epsilon$ for any $\epsilon >0$. $\square$

### Statistical significance of the results

To compute a threshold that guarantees statistical confidence of our finding, we first compute a bound on the gap in a non significant set.

#### **Theorem 2**

*Assume that* *S* *is not a significant set, i.e.,* ${\mathcal {C}}$ *and* ${\mathcal {D}}$ *have the same distribution on* *S,* *then*

$$\begin{aligned} Prob( dc_{S}({\mathcal {C}},{\mathcal {D}}) > \epsilon )\le 2e^{-2 \epsilon ^2 n_{{\mathcal {C}}}n_{{\mathcal {D}}}/(n_{{\mathcal {C}}}+n_{{\mathcal {D}}})}. \end{aligned}$$

#### *Proof*

Let $X_1,\dots , X_{n_C}$ be independent random variables such that $X_i=1/n_C$ if sample $c_i$ in ${\mathcal {C}}$ has a mutation in *S*, otherwise $X_i=0$. Similarly, let $Y_1,\dots , Y_{n_D}$ be independent random variables such that $Y_i= -1/n_D$ if sample $d_i$ in ${\mathcal {D}}$ has a mutation in *S*, otherwise $Y_i=0$.

Clearly $dc_{S}({\mathcal {C}},{\mathcal {D}}) = \sum _{i=1}^{n_C} X_i + \sum _{i=1}^{n_D} Y_i$, and since *S* is not significant $E\left[\sum _{i=1}^{n_C} X_i +\sum _{i=1}^{n_D} Y_i\right]=0$.

To apply Hoeffding bound (Theorem 4.14 in [29]), we note that the sum $\sum _{i=1}^{n_C} X_i + \sum _{i=1}^{n_D} Y_i$ has $n_C$ variables in the range $[0,1/n_C]$, and $n_D$ variables in the range $[-1/n_D, 0]$. Thus,

$$\begin{aligned} Prob( dc_{S}({\mathcal {C}},{\mathcal {D}}) > \epsilon )\le 2e^{(-2 \epsilon ^2 )/(n_c/n_c^2 + n_d/n_D^2)} = 2e^{-2 \epsilon ^2 n_{{\mathcal {C}}}n_{{\mathcal {D}}}/(n_{{\mathcal {C}}}+n_{{\mathcal {D}}})}. \end{aligned}$$

$$\square$$

Let $N_{k}$ be the set of subnetworks under consideration, or the set of all connected components of size $\le k$. We use Theorem 2 to obtain guarantees on the statistical significance of the results of DAMOKLE  in terms of the Family-Wise Error Rate (FWER) or of the False Discovery Rate (FDR) as follows:

- FWER: if we want to find just the subnetwork with significant maximum differential coverage, to bound the FWER of our method by $\alpha$ we use the maximum $\epsilon$ such that $N_{k} 2e^{-2 \epsilon ^2 n_{{\mathcal {C}}}n_{{\mathcal {D}}}/(n_{{\mathcal {C}}}+n_{{\mathcal {D}}})}\le \alpha .$
- FDR: if we want to find several significant subnetworks with high differential coverage, to bound the FDR by $\alpha$ we use the maximum $\epsilon$ such that ${N_{k} 2e^{-2 \epsilon ^2 n_{{\mathcal {C}}}n_{{\mathcal {D}}}/(n_{{\mathcal {C}}}+n_{{\mathcal {D}}})}}/n(\alpha ) \le \alpha$, where $n(\alpha )$ is the number of sets with differential coverage $\ge \epsilon$.

### Permutation testing

While Theorem 2 shows how to obtain guarantees on the statistical significance of the results of DAMOKLE by appropriately setting $\theta$, in practice, due to relatively small sample sizes and to inevitable looseness in the theoretical guarantees, a permutation testing approach may be more effective in estimating the statistical significance of the results of DAMOKLE and provide more power for the identification of differentially mutated subnetworks.

We consider two permutation tests to assess the association of mutations in the subnetwork with the highest differential coverage found by DAMOKLE. The first test assesses whether the observed differential coverage can be obtained under the independence of mutations in genes by considering the null distribution in which each gene is mutated in a random subset (of the same cardinality as observed in the data) of all samples, independently of all other events. The second test assesses whether, under the observed marginal distributions for mutations in sets of genes, the observed differential coverage of a subnetwork can be obtained under the independence between mutations and samples’ memberships (i.e., being a sample of ${\mathcal {C}}$ or a sample of ${\mathcal {D}}$), by randomly permuting the samples memberships.

Let $dc_{S}({\mathcal {C}},{\mathcal {D}})$ be the differential coverage observed on real data for the solution *S* with highest differential coverage found by DAMOKLE (for some input parameters). For both tests we estimate the *p*-value as follow:

1. generate *N* (permuted) datasets from the null distribution;
2. run DAMOKLE (with the same input parameters used on real data) on each of the *N* permuted datasets;
3. let *x* be the number of permuted datasets in which DAMOKLE reports a solution with differential coverage $\ge dc_{S}({\mathcal {C}},{\mathcal {D}})$: then the *p*-value of *S* is $(x+1)/(N+1)$.

## Results

We implemented DAMOKLE in Python^1^ and tested it on simulated and on cancer data. Our experiments have been conducted on a Linux machine with 16 cores and 256 GB of RAM. For all our experiments we used as interaction graph *G* the HINT+HI2012 network^2^, a combination of the HINT network [30] and the HI-2012  [31] set of protein–protein interactions, previously used in [5]. In all cases we considered only the subnetwork with the highest differential coverage among the ones returned by DAMOKLE. We first present the results on simulated data ("Simulated data" section) and then present the results on cancer data ("Cancer data" section).

### Simulated data

We tested DAMOKLE on simulated data generated as follows. We assume there is a subnetwork *S* of *k* genes with differential coverage $dc_{S}({\mathcal {C}},{\mathcal {D}})= c$. In our simulations we set $|{\mathcal {C}}|=|{\mathcal {D}}|=n$. For each sample in ${\mathcal {D}}$, each gene *g* in *G* (including genes in *S*) is mutated with probability $p_g$, independently of all other events. For samples in ${\mathcal {C}}$, we first mutated each gene *g* with probability $p_g$ independently of all other events. We then considered the samples of ${\mathcal {C}}$ without mutations in *S*, and for each such sample we mutated, with probability *c*, one gene of *S*, chosen uniformly at random. In this way *c* is the *expectation* of the differential coverage $dc_{S}({\mathcal {C}},{\mathcal {D}})$. For genes in $G \setminus S$ we used mutation probabilities $p_g$ estimated from oesophageal cancer data [32]. We considered only value of $n \ge 100$, consistent with sample sizes in most recent cancer sequencing studies. (The latest ICGC data release^3^ from April 30$^{th}$, 2018 has data for $\ge 500$ samples for $81\%$ of the primary sites).

The goal of our investigation using simulated data is to evaluate the impact of various parameters on ability of DAMOKLE to recover *S* or part of it. In particular, we studied the impact of three parameters: the differential coverage $dc_{S}({\mathcal {C}},{\mathcal {D}})$ of the planted subnetwork *S*; the number *k* of genes in *S*; and the number *n* of samples in each class. To evaluate the impact of such parameters, for each combination of parameters in our experiments we generated 10 simulated datasets and run DAMOKLE on each dataset with $\theta = 0.01$, recording

1. the fraction of times that DAMOKLE reported *S* as the solution with the highest differential coverage, and
2. the fraction of genes of *S* that are in the solution with highest differential coverage found by DAMOKLE.

**Fig. 2:**
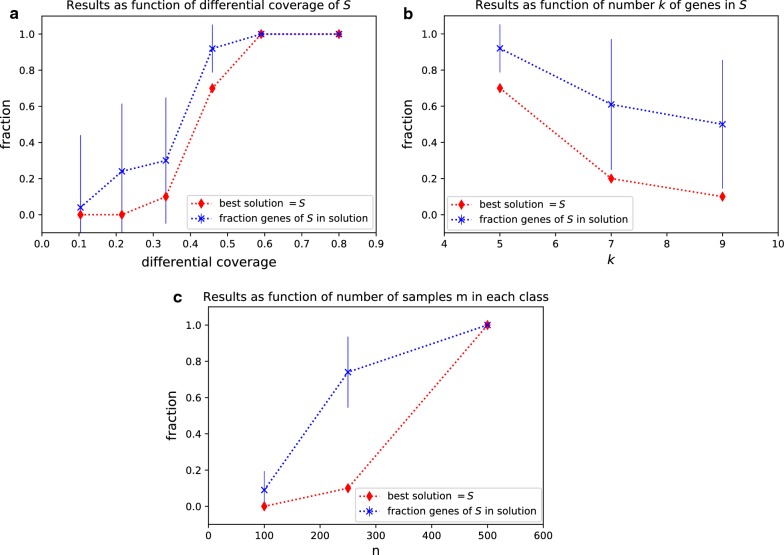
**a** Performance of DAMOKLE as a function of the differential coverage $dc_{S}({\mathcal {C}},{\mathcal {D}})$ of subnetwork *S*. The figure shows (red) the fraction of times, out of 10 experiments, that the best solution corresponds to *S* and (blue) the fraction of genes in *S* that are reported in the best solution by DAMOKLE. For the latter, error bars show the standard deviation on the 10 experiments. $n=100$ and $k=5$ for all experiments. **b** Performance of DAMOKLE as a function of the number *k* of genes in subnetwork *S*. $n=100$ and $dc_{S}({\mathcal {C}},{\mathcal {D}})=0.46$ for all experiments. **c** Performance of DAMOKLE as a function of the number *n* of samples in ${\mathcal {C}},{\mathcal {D}}$. $k=10$ and $dc_{S}({\mathcal {C}},{\mathcal {D}})=0.46$ for all experiments

We first investigated the impact of the differential coverage $c = dc_{S}({\mathcal {C}},{\mathcal {D}})$. We analyzed simulated datasets with $n=100$ samples in each class, where $k=5$ genes are part of the subnetwork *S*, for values of $c = 0.1, 0.22, 0.33, 0.46, 0.6, 0.8$,. We run DAMOKLE on each dataset with $k=5$. The results are shown in Fig. 2a. For low values of the differential coverage *c*, with $n=100$ samples DAMOKLE never reports *S* as the best solution found and only a small fraction of the genes in *S* are part of the solution reported by DAMOKLE. However, as soon as the differential coverage is $\ge 0.45$, even with $n=100$ samples in each class DAMOKLE identifies the entire planted solution *S* most of the times, and even when the best solution does not entirely corresponds to *S*, more than $80\%$ of the genes of *S* are reported in the best solution. For values of $c \ge 0.6$, DAMOKLE *always* reports the whole subnetwork *S* as the best solution. Given that many recent large cancer sequencing studies consider at least 200 samples, DAMOKLE will be useful to identify differentially mutated subnetworks in such studies.

We then tested the performance of DAMOKLE as a function of the number of genes *k* in *S*. We tested the ability of DAMOKLE to identify a subnetwork *S* with differential coverage $dc_{S}({\mathcal {C}},{\mathcal {D}})=0.46$ in a dataset with $n=100$ samples in both ${\mathcal {C}}$ and ${\mathcal {D}}$, when the number *k* of genes in *S* varies as $k=5,7,9$. The results are shown in Fig. 2b. As expected, when the number of genes in *S* increases, the fraction of times *S* is the best solution as well as the fraction of genes reported in the best solution by *S* decreases, and for $k=9$ the best solution found by DAMOKLE corresponds to *S* only $10\%$ of the times. However, even for $k=9$, on average most of the genes of *S* are reported in the best solution by DAMOKLE. Therefore DAMOKLE can be used to identify relatively large subnetworks mutated in a significantly different number of samples even when the number of samples is relatively low.

Finally, we tested the performance of DAMOKLE as the number of samples *n* in each set ${\mathcal {C}},{\mathcal {D}}$ increases. In particular, we tested the ability of DAMOKLE to identify a relatively large subnetwork *S* of $k=10$ genes with differential coverage $dc_S({\mathcal {C}},{\mathcal {D}}) = 0.46$ as the number of samples *n* increases. We analyzed simulated datasets for $n=100, 250, 500$. The results are shown in Fig. 2. For $n=100$, when $k=10$, DAMOKLE never reports *S* as the best solution and only a small fraction of all genes in *S* are reported in the solution. However, for $n=250$, while DAMOKLE still reports *S* as the best solution only $10\%$ of the times, on average $70\%$ of the genes of *S* are reported in the best solution. More interestingly, already for $n=500$, DAMOKLE *always* reports *S* as the best solution. These results show that DAMOKLE can reliably identify relatively large differentially mutated subnetworks from currently available datasets of large cancer sequencing studies.

### Cancer data

We use DAMOKLE to analyze somatic mutations from The Cancer Genome Atlas. We first compared two similar cancer types and two very different cancer types to test whether DAMOKLE behaves as expected on these types. We then analyzed two pairs of cancer types where differences in alterations are unclear. In all cases we run DAMOKLE with $\theta =0.1$ and obtained *p*-values with the permutation tests described in "Permutation testing" section.

#### Lung cancer

We used DAMOKLE to analyze 188 samples of lung squamous cell carcinoma (LUSC) and 183 samples of lung adenocarcinoma (LUAD). We only considered single nucleotide variants (SNVs)^4^ and use $k=5$. DAMOKLE did not report any significant subnetwork, in agreement with previous work showing that these two cancer types have known differences in gene expression [33] but are much more similar with respect to SNVs [34].

#### Colorectal vs ovarian cancer

We used DAMOKLE to analyze 456 samples of colorectal adenocarcinoma (COADREAD) and 496 samples of ovarian serous cystadenocarcinoma (OV) using only SNVs.^5^ For $k=5$, DAMOKLE identifies the significant ($p<0.01$ according to both tests in "Permutation testing" section) subnetwork APC, CTNNB1, FBXO30, SMAD4, SYNE1 with differential coverage 0.81 in COADREAD w.r.t. OV. APC, CTNNB1, and SMAD4 are members of the WNT signaling and TFG-$\beta$ signaling pathways. The WNT signaling pathway is one of the cascades that regulates stemness and development, with a role in carcinogenesis that has been described mostly for colorectal cancer [35], but altered Wnt signaling is observed in many other cancer types [36]. The TFG-$\beta$ signaling pathway is involved in several processes including cell growth and apoptosis, that is deregulated in many diseases, including COADREAD [35]. The high differential coverage of the subnetwork is in accordance with COADREAD being altered mostly by SNVs and OV being altered mostly by copy number aberrations (CNAs) [37].

#### Esophagus-stomach cancer

**Fig. 3:**
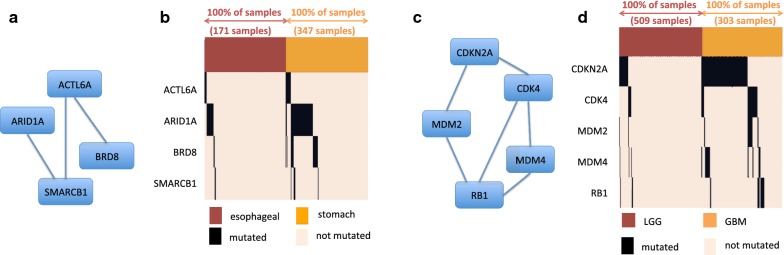
Results of DAMOKLE analysis of esophagus tumours and stomach tumours and of diffuse gliomas. **a** Subnetwork *S* with significant ($p<0.01$) differential coverage in esophagus tumours vs stomach tumours (interactions from HINT+HI2012 network). **b** Fractions of samples with mutations in genes of *S* in esophagus tumours and in stomach tumours. **c** Subnetwork *S* with significant ($p<0.01$) differential coverage in LGG samples vs GBM samples (interactions from HINT+HI2012 network). **d** Fractions of samples with mutations in genes of *S* in LGG samples and GBM samples

We analyzed SNVs and CNAs in 171 samples of esophagus cancer and in 347 samples of stomach cancer [32].^6^ The number of mutations in the two sets is not significantly different (t-test *p* = 0.16). We first considered single genes, identifying TP53 with high ($>0.5$) differential coverage between the two cancer types. Alterations in TP53 have then be removed for the subsequent DAMOKLE analysis. We run DAMOKLE with $k=4$ with ${\mathcal {C}}$ being the set of stomach tumours and ${\mathcal {D}}$ being the set of esophagus tumours. DAMOKLE identifies the significant ($p<0.01$ for both tests in "Permutation testing" section) subnetwork $S=$ {ACTL6A, ARID1A, BRD8, SMARCB1} with differential coverage 0.26 (Fig. 3a, b). Interestingly, all four genes in the subnetwork identified by DAMOKLE are members of the chromatin organization machinery recently associated with cancer [38, 39]. Such subnetwork is not reported as differentially mutated in the TCGA publication comparing the two cancer types [32]. BRD8 is only the top-16 gene by differential coverage, while ACTL6 and SMARCB1 are not among the top-2000 genes by differential coverage. We compared the results obtained by DAMOKLE with the results obtained by HotNet2 [5], a method to identify significantly mutated subnetworks, using the same mutation data and the same interaction network as input: none of the genes in *S* appeared in significant subnetworks reported by HotNet2.

#### Diffuse gliomas

We analyzed single nucleotide variants (SNVs) and copy number aberrations (CNAs) in 509 samples of lower grade glioma (LGG) and in 303 samples of glioblastoma multiforme (GBM).^7^ We considered nonsilent SNVs, short indels, and CNAs. We removed from the analysis genes with $<6$ mutations in both classes. By single gene analysis we identified IDH1 with high ($>0.5$) differential coverage, and removed alterations in such gene for the DAMOKLE analysis. We run DAMOKLE with $k=5$ with ${\mathcal {C}}$ being the set of GBM samples and ${\mathcal {D}}$ being the set of LGG samples. The number of mutations in ${\mathcal {C}}$ and in *D* is not significantly different (t-test *p* = 0.1). DAMOKLE identifies the significant ($p<0.01$ for both tests in "Permutation testing" section) subnetwork $S=$ {CDKN2A, CDK4, MDM2, MDM4, RB1} (Fig. 3c, d). All genes in *S* are members of the p53 pathway or of the RB pathway. The p53 pathway has a key role in cell death as well as in cell division, and the RB pathway plays a crucial role in cell cycle control. Both pathways are well known glioma cancer pathways [40]. Interestingly, [41] did not report any subnetwork with significant difference in mutations between LGG and GBM samples. CDK4, MDM2, MDM4, and RB1 do not appear among the top-45 genes by differential coverage. We compared the results obtained by DAMOKLE with the results obtained by HotNet2. Of the genes in our subnetwork, only CDK4 and CDKN2A are reported in a significantly mutated subnetwork ($p <0.05$) obtained by HotNet2 analyzing ${\mathcal {D}}$ but not analyzing ${\mathcal {C}}$, while MDM2, MDM4, and RB1 are not reported in any significant subnetwork obtained by HotNet2.

## Conclusion

In this work we study the problem of finding subnetworks of a large interaction network with significant difference in mutation frequency in two sets of cancer samples. This problem is extremely important to identify mutated mechanisms that are specific to a cancer (sub)type as well as for the identification of mechanisms related to clinical features (e.g., response to therapy). We provide a formal definition of the problem and show that the associated computational problem is NP-hard. We design, analyze, implement, and test a simple and efficient algorithm, DAMOKLE, which we prove identifies significant subnetworks when enough data from a reasonable generative model for cancer mutations is provided. Our results also show that the subnetworks identified by DAMOKLE cannot be identified by methods not designed for the *comparative* analysis of mutations in two sets of samples. We tested DAMOKLE on simulated and real data. The results on simulated data show that DAMOKLE identifies significant subnetworks with currently available sample sizes. The results on two large cancer datasets, each comprising genome-wide measurements of DNA mutations in two cancer subtypes, shows that DAMOKLE identifies subnetworks that are not found by methods not designed for the *comparative* analysis of mutations in two sets of samples.

While we provide a first method for the differential analysis of cohorts of cancer samples, several research directions remain. First, differences in the frequency of mutation of a subnetwork in two sets of cancer cohorts may be due to external (or hidden) variables, as for example the mutation rate of each cohort. While at the moment we ensure before running the analysis that no significant difference in mutation rate is present between the two sets, performing the analysis while correcting for possible differences in such confounding variable or in others would greatly expand the applicability of our method. Second, for some interaction networks (e.g., functional ones) that are relatively more dense than the protein–protein interaction network we consider, requiring a minimum connectivity (e.g., in the form of fraction of all possible edges) in the subnetwork may be beneficial, and the design of efficient algorithms considering such requirement is an interesting direction of research. Third, different types of mutation patterns (e.g., mutual exclusivity) among two set of samples could be explored (e.g., extending the method proposed in [42]). Fourth, the inclusion of additional types of measurements, as for example gene expression, may improve the power of our method. Fifth, the inclusion of noncoding variants in the analysis may provide additional information to be leveraged to assess the significance of subnetworks.

## Footnotes

1 The implementation is available at https://github.com/VandinLab/DAMOKLE.

2 http://compbio-research.cs.brown.edu/pancancer/hotnet2/.

3 https://dcc.icgc.org/.

4 http://cbio.mskcc.org/cancergenomics/pancan_tcga/.

5 http://cbio.mskcc.org/cancergenomics/pancan_tcga/.

6 http://www.cbioportal.org/study?id=stes_tcga_pub#summary.

7 https://media.githubusercontent.com/media/cBioPortal/datahub/master/public/lgggbm_tcga_pub.tar.gz.
